# A new Ecuadorian species of the rare Neotropical caddisfly genus *Amphoropsyche* Holzenthal (Trichoptera, Leptoceridae)

**DOI:** 10.3897/zookeys.640.10344

**Published:** 2016-12-13

**Authors:** Ralph W. Holzenthal, Blanca Ríos-Touma

**Affiliations:** 1 University of Minnesota, Department of Entomology, 1980 Folwell Ave., 219 Hodson Hall, St. Paul, Minnesota 55108 U.S.A.; 2 Universidad de las Américas, Facultad de Ingenierías y Ciencias Agropecuarias, Unidad de Investigación en Biotecnología y Medio Ambiente -BIOMA-, Campus Queri, Calle José Queri, Edificio #8, PB, Quito, Ecuador

**Keywords:** Trichoptera, caddisfly, Neotropics, new species, key to species, rare, endemic, Andes

## Abstract

A new species of the rare long-horned caddisfly genus *Amphoropsyche* Holzenthal is described from Ecuador, bringing the number of species known from the genus to 15. All species are very regional in their distributions and known only from very few specimens. The new species, *Amphoropsyche real*, is similar to a number of previously described species from Colombia (*Amphoropsyche ayura*, *Amphoropsyche cauca*, *Amphoropsyche flinti*, *Amphoropsyche quebrada*, and *Amphoropsyche stellata*) and Ecuador (*Amphoropsyche napo* and *Amphoropsyche tandayapa*). The males can be distinguished from the others by features of segment X of the male genitalia, especially the prominent midlateral and subapicodorsal spinelike setae. An updated taxonomic key to males of the genus is provided.

## Introduction

The long-horned caddisfly genus *Amphoropsyche* Holzenthal, 1985 is endemic to the Neotropical Region where it now contains 15 described species (Table 1), including a new species described herein. While widely distributed, its species are among the rarest of the Neotropical caddisflies. Only a few specimens are known to exist in museum collections and almost all of the specimens known to us are housed in 2 insect drawers, 1 at the University of Minnesota and 1 at the Smithsonian Institution. Whether the species are habitat specialists or do not fly to UV light traps set at dusk (the most common method for collecting adult caddisflies) is unknown. On one occasion, the senior author netted individuals of *Amphoropsyche woodruffi woodruffi* Flint and Sykora, swarming during mid-day over a small, shallow stream in Venezuela. Otherwise, they are collected 1 or 2 at a time from lights or from Malaise traps. Larvae are known only for one species, *Amphoropsyche insularis* (Flint), recorded from the sandy bottom of a pool in a small stream on the Lesser Antillean island of Dominica. Larvae build cases of sand grains (Holzenthal 1986). Adults of the other species also seem to be associated with small, gravel-bottomed streams in hilly or mountainous areas. Adult males contain interesting pheromone dispersing structures on the genitalia (Botosaneanu 1991). In this paper we describe the 15th known species in the genus and the 3rd recorded from Ecuador. The new species is known from 1 male and 2 female specimens. The previously most recently described species, *Amphoropsyche tandyapa* Holzenthal and Rázuri-Gonzales, also from Ecuador, is known from only the male holotype. These discoveries further corroborate Holzenthal’s (1986) prediction that the northern Andes harbor a rich fauna of these very rare, enigmatic icons of the Neotropical caddisfly fauna.

**Table 1.** Species, distributions, and published records of known individuals in the caddisfly genus *Amphoropsyche* (Leptoceridae). HT = holotype, PT = paratype, * = immature stages known.

Species	Distribution	Known individuals in literature	Additional references
***aragua*** Holzenthal, 1985	Venezuela	male HT, 5 male PTs	–
***ayura*** Holzenthal, 1985	Colombia	male HT, 1 female PT	Flint 1991
***cauca*** Holzenthal, 1985	Colombia	male HT	Flint 1991
***choco*** Holzenthal, 1985	Colombia	male HT	–
***flinti*** Holzenthal, 1985	Colombia	male HT	Flint 1991
***insularis*** (Flint, 1968) *Brachysetodes* *	Dominica, Guadeloupe, Martinique	male HT, 48 male, 21 female PTs, 3 larvae, 1 pupa; 4 additional specimens	Holzenthal 1985, 1986; Flint and Sykora 1993; Botosaneanu 1994; Botosaneanu and Thomas 2005
***janstockiana*** Botosaneanu, 1990	St. Vincent, Mustique[?]	male HT, 3 male PTs	Botosaneanu 1991; Flint and Sykora 1993
***napo*** Holzenthal, 1985	Ecuador	male HT	–
***quebrada*** Holzenthal, 1985	Colombia	male HT, 2 female PTs	Flint 1991
***real* Holzenthal & Ríos-Touma, sp. n.**	Ecuador	male HT, 2 female PTs	–
***refugia*** Holzenthal, 1985	Venezuela	male HT, 1 male, 1 female PTs	–
***spinifera*** Holzenthal, 1986	Bolivia, Peru	male HT, 3 male, 1 female PTs	Flint 1996
***stellata*** Holzenthal, 1985	Colombia	male HT	–
***tandayapa*** Holzenthal & Rázuri-Gonzales, 2011	Ecuador	male HT	–
***woodruffi multispinosa*** Botosaneanu, 1993, in Botosaneanu & Alkins-Koo, 1993	Trinidad	male HT, 3 female PTs; 9 additional specimens	Botosaneanu and Sakal 1992; Flint 1996
***woodruffiwoodruffi*** Flint & Sykora, 1993	Grenada, Venezuela	male HT; 40 additional specimens	Flint 1996

## Material and methods

This species is based on material collected by the authors and their colleagues during an ongoing inventory of the Trichoptera of Ecuador. Specimens were attracted to a UV light placed over a shallow, white plastic pan filled with 80% ethyl alcohol placed next to a small, gravel stream. Techniques and procedures used in the preparation and examination of the specimen were outlined by Blahnik and Holzenthal (2004) and Blahnik et al. (2007). The illustrations of the genitalia were prepared from pencil sketches made with the aid of a drawing tube mounted on an Olympus BX41 compound microscope. The pencil sketches were then scanned and placed into an Adobe Illustrator (version CC, Adobe Systems, Inc.) document, to serve as a template, and then traced to create a vector graphic illustration. A graphic tablet and pen (Intuous^TM^, Wacom Technology Co.) facilitated careful tracing of the original image.

Terminology used in describing male and female genitalia follows that of Holzenthal (1985, 1986). The updated taxonomic key is modified from that published by Holzenthal and Rázuri-Gonzales (2011) and is based on published illustrations and descriptions of the male genitalia (Holzenthal 1985, 1986; Botosaneanu 1990; Flint and Sykora 1993, Botosaneanu and Alkins-Koo 1993; Flint 1996 [these papers can be downloaded from the *Trichoptera Literature Database* at www.trichopteralit.umn.edu to facilitate comparisons]). The types are deposited in the University of Minnesota Insect Collection (UMSP), St. Paul, Minnesota, USA, and the Museo Ecuatoriano de Ciencias Naturales (MECN), Quito, Ecuador. The specimens are preserved in 80% ethyl alcohol.

## Systematics

### Genus Amphoropsyche

Taxon classificationAnimaliaTrichopteraLeptoceridae

Holzenthal

Amphoropsyche Holzenthal, 1985:255 [Type species: Brachysetodes insularis Flint, 1968, original designation]. —Holzenthal 1985:254 [revision]. —Holzenthal 1986:251 [larva, pupa]. —Holzenthal and Rázuri-Gonzales 2011:63 [key to species].

### Amphoropsyche real

Taxon classificationAnimaliaTrichopteraLeptoceridae

Holzenthal & Ríos-Touma sp. n.

http://zoobank.org/4D4D3E54-69B4-4E43-A8BE-3052E60F1F95

Figs 1 , 2

#### Diagnosis.

This new species is most similar to *Amphoropsyche napo* and *Amphoropsyche tandayapa*, from Ecuador, and the Colombian species *Amphoropsyche ayura*, *Amphoropsyche cauca*, *Amphoropsyche flinti*, *Amphoropsyche quebrada*, and *Amphoropsyche stellata*. All of these species share tergum X bearing a mesal process and paired, lateral processes of various forms. The new species is the only one with the combination of long, spatulate mesal process and the lateral processes bearing both a prominent midlateral and a prominent subapicodorsal spinelike seta. In addition, *Amphoropsyche ayura*, *Amphoropsyche napo*, *Amphoropsyche quebrada*, *Amphoropsyche stellata*, and *Amphoropsyche tandayapa* have prominent parameres in the phallus, lacking in *Amphoropsyche real*, sp. n., while *Amphoropsyche cauca*, *Amphoropsyche flinti*, and *Amphoropsyche tandayapa* have a baso- or mesoventral process on the inferior appendages not present in the new species.

#### Description.

**Figure 1.**
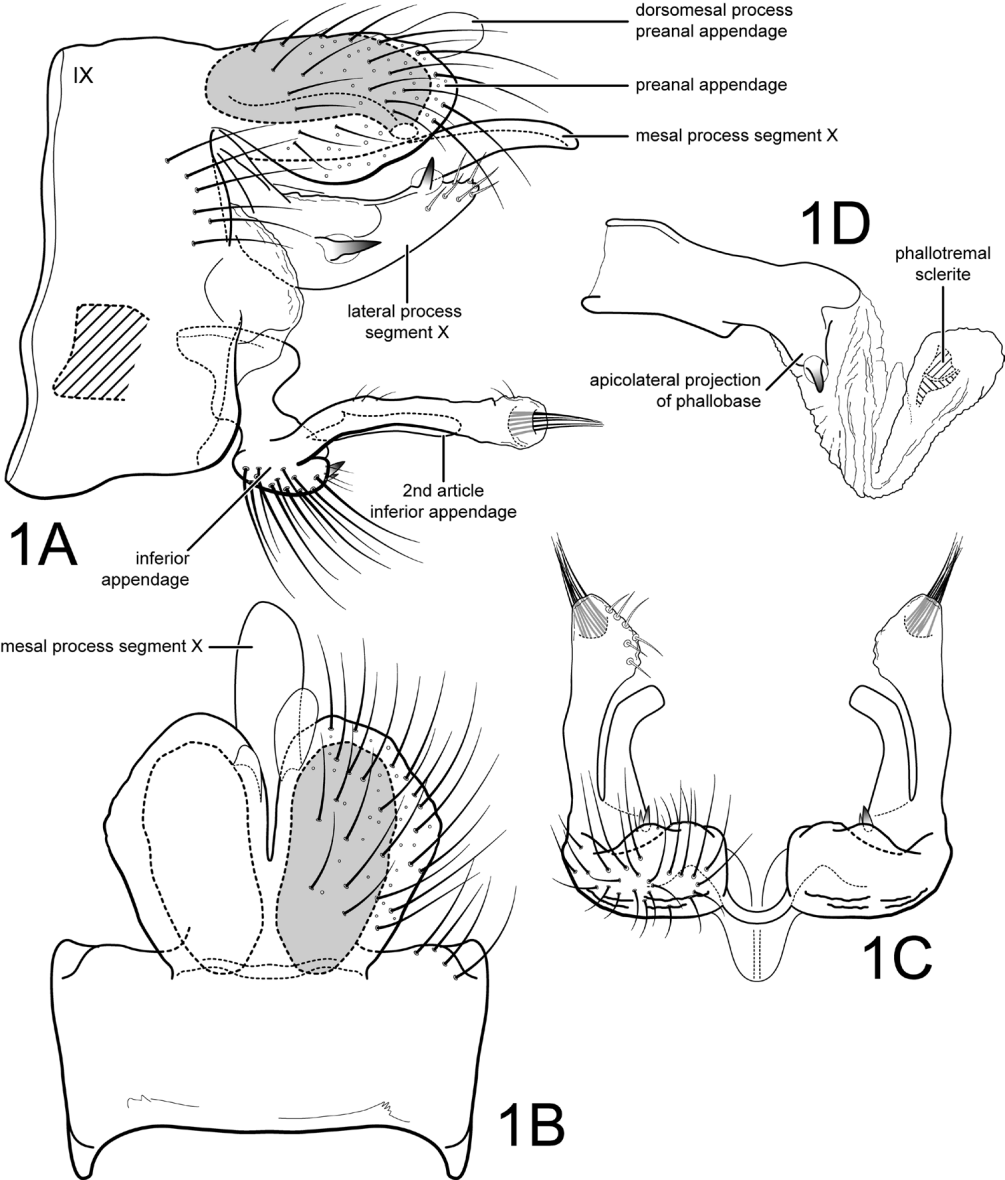
*Amphoropsyche real*, new species. Male genitalia **A** lateral **B** segments IX-X, dorsal **C** inferior appendages, ventral **D** phallus, lateral. IX = abdominal segment IX.

Male. Forewing length 5.0 mm. Body and legs stramineous, wings light brown, apical 1/5th light cream (specimen preserved in 80% ethyl alcohol). Genitalia as in Fig. 1A–D. Segment IX annular, sternum with anterior part not extended anteriorly (Fig. 1A). Segment X composed of a single mesal process and pair of lateral processes (Fig. 1A–B); mesal process long, spatulate, apex rounded (Fig. 1B); lateral process broadly crescent shaped, bearing large lateral spinelike seta at midlength and large subapicodorsal spinelike seta; apically lateral process with about 6-8 small, but prominent setae (Fig. 1A). Preanal appendages large, oval, fused basally but divided apically to 1/2 their lengths (apical emargination acute); with large reticulate internal gland and small subapicoventral pore (Fig. 1A–B); apically with pair of asymmetrical, membranous dorsomesal processes, left process large bulbous, right process short (Fig. 1B). [It is highly likely that this asymmetry was caused by a malformation of one or the other or both processes. These processes may be prone to developmental abnormalities.] Inferior appendage with 1st article narrow, elongate, without basoventral projection, instead base short, bulbous and at right angle to straight apical portion of 1st article when viewed ventrally, bearing 2 small spinelike setae on posterior face (Fig. 1A, C); 1st article ending in a bulbous apex, bearing subterminal tuft of closely appressed setae emerging from membranous pocket; 2nd article of inferior appendage elongate, thin, slightly curved inwards in ventral view, apex slightly truncate; 2nd article fused to 1st article at base (or articulation not apparent) (Fig. 1C). Phallic apparatus (Fig. 1D) with phallobase well developed, with sclerotized apicolateral projection on each side, bearing stout apical spine; parameres absent; endothecal membranes well developed, apparently capable of articulation at midlength [these membranes were evaginated by the clearing process]; phallotremal sclerite well developed, structure as illustrated in Fig. 1D, but difficult to discern on specimen.

**Figure 2.**
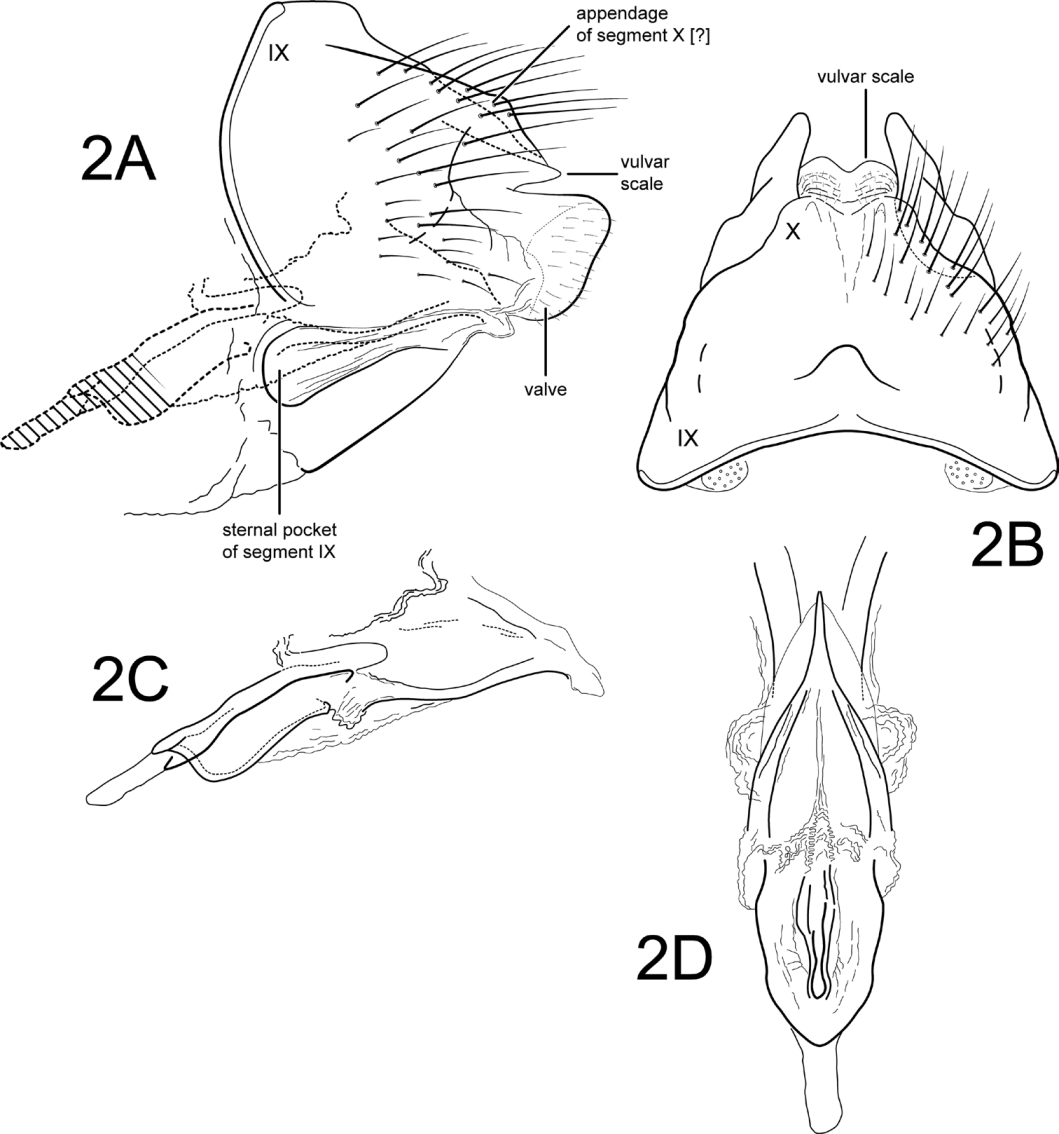
*Amphoropsyche real*, new species. Female genitalia **A** lateral **B** segments IX-X, dorsal **C** vaginal apparatus, lateral **D** vaginal apparatus, ventral. IX = abdominal segment IX, X = abdominal segment X.

Female. Forewing length 6.0 mm (n=2). Color and structure similar to male’s (specimens preserved in 80% ethyl alcohol). Genitalia as in Fig. 2A–D. Abdominal tergum IX + X very slightly excised apicomesally, tergum basally with small moundlike mesal protuberance; dorsomesally slightly excised along length. Appendages of segment X absent (or highly reduced), apparent only as slightly raised, dorsolateral setose areas. Valves posterolateral, quadrate, covered with short setae. Vulvar scale thin, narrow in lateral view, round in dorsal view with slight mesal excavation. Sternum IX laterally forming pocketlike structure in pleural region (probably receptacle for apex of male inferior appendage). Vaginal apparatus (spermathecal sclerite complex) (see Fig. 2C, D) with broad, posterior base bearing central “keyhole-shaped” structure; middle region to apex with narrow lightly sclerotized plates and 2 dorsal membranous rounded mounds.

**Holotype:** **Male.** **ECUADOR: Morona-Santiago:** Macas, small gravel stream (Wallace/Real property), 02.20299°S, 078.08539°W, el. 1076 m, 27.i.2015, Holzenthal, Huisman, Ríos-Touma, Amigo (UMSP000114167) (UMSP). **Paratypes:** same data as holotype, 2 females (UMSP, MECN).

#### Etymology.

Named for the family of RhoAnn Wallace and Galo Real and their children, Aster, Diem, and Luna, in recognition of their hospitality, friendship, and stewardship of the land where this species was collected.

### Revised key to males of *Amphoropsyche* (modified from Holzenthal and Rázuri-Gonzales 2011)

**Table:** 

1	Preanal appendages completely (Holzenthal 1985, figs 8B, 10B; Flint and Sykora 1993, fig. 20) or almost completely fused medially (if the latter, apical emargination shallow, obtuse) (Holzenthal and Rázuri-Gonzales 2011, fig. 1B; Holzenthal 1985, fig. 3B)	**2**
–	Preanal appendages not fused medially, divided to 1/3 to 2/3 of their length (apical emargination acute) (Fig. 1B; Holzenthal 1985, figs 5B, 6B)	**6**
2(1)	Preanal appendages with dorsomesal process or processes (Holzenthal 1985, figs 8B, 10B)	**3**
–	Preanal appendages without dorsomesal process or processes (Holzenthal 1985, figs 3A–D)	***Amphoropsyche insularis***
3(2)	Dorsomesal processes of preanal appendages very short, digitate, not exceeding length of preanal appendage (Holzenthal and Rázuri-Gonzales 2011, figs 1A–D); dorsomesal processes of preanal appendages not sclerotized	***Amphoropsyche tandayapa***
–	Dorsomesal process or processes of preanal appendages long, ca. length of preanal appendage (Holzenthal 1985, figs 8A, 10A; Flint and Sykora 1993, fig. 18); dorsomesal processes of preanal appendages sclerotized	**4**
4(3)	Second article of inferior appendages elongate, narrow (Holzenthal 1985, fig. 8A)	**5**
–	Second article of inferior appendages short (Flint and Sykora 1993, figs 18–20; Botosaneanu and Alkins-Koo 1993, figs 97–101)	***Amphoropsyche woodruffi***
5(4)	Dorsomesal process of preanal appendages bifid in dorsal view; ventral subterminal portion of phallobase serrate (Holzenthal 1985, figs 8A–D)	***Amphoropsyche refugia***
–	Dorsomesal process of the preanal appendages entire in dorsal view; ventral subterminal portion of phallobase entire (Holzenthal 1985, figs 10A–D)	***Amphoropsyche aragua***
6(1)	Second article of inferior appendages present (Holzenthal 1985, fig. 5A)	**7**
–	Second article of inferior appendages absent (Holzenthal 1985, fig. 16C)	**14**
7(6)	Tergum X with mesal process and paired lateral processes (Holzenthal 1985, figs 5A, 14A)	**8**
–	Tergum X without mesal process, lateral processes with apical and subapical spinelike projections (Botosaneanu 1990, figs 1–3)	***Amphoropsyche janstockiana***
8(7)	Second article of inferior appendages short (Holzenthal 1985, fig. 14C) or long, but broad (Holzenthal 1985, fig. 6C)	**9**
–	Second article of inferior appendages elongate and narrow (Holzenthal 1985, Fig. 7C)	**11**
9(8)	Phallus without parameres (Holzenthal 1985, fig. 6D)	**10**
–	Phallus with parameres (Holzenthal 1985, figs 14A–D)	***Amphoropsyche quebrada***
10(9)	Second article of inferior appendages short, with apical spine-like seta; lateral process of tergum X with subapical spine-like seta; phallicata with pair of bifid, spiniferous, lateral extensions (Holzenthal 1986, figs 1A–D)	***Amphoropsyche spinifera***
–	Second article of inferior appendages long, but broad, without apical spinelike seta; lateral process of tergum X with several apical spine-like setae; phallicata without lateral, bifid extensions, but phallobase with ventral spinelike process (Holzenthal 1985, figs 6A–D)	***Amphoropsyche flinti***
11(8)	Phallus with parameres (Holzenthal 1985, fig. 5D) or phallobase with sclerotized apical projection bearing a stout spine	**12**
–	Phallus without parameres (Holzenthal 1985, figs 11A–D)	***Amphoropsyche choco***
12(11)	Inferior appendage with a prominent (Holzenthal 1985, figs 5A, C) to short mesoventral lobe (Holzenthal 1985, fig. 7C) when viewed ventrally; phallus with parameres (Holzenthal 1985, figs 5D, 7D)	**13**
–	Inferior appendage without mesoventral lobe (Fig. 1C); phallus without parameres, but phallobase with sclerotized apical projection bearing a stout spine (Fig. 1)	***Amphoropsyche real* sp. n.**
13(12)	Lateral process of tergum X U-shaped, tip bifid, bearing small spinelike setae (Holzenthal 1985, figs 5A–D)	***Amphoropsyche napo***
–	Lateral process of tergum X tapered to a sharp terminal point, without spinelike setae (Holzenthal 1985, figs 7A–D)	***Amphoropsyche stellata***
14(6)	Parameres small; inferior appendage with basoventral lobe (Holzenthal 1985, figs 16A–D)	***Amphoropsyche cauca***
–	Parameres large; inferior appendage without basoventral lobe (Holzenthal 1985, figs 12A–D)	***Amphoropsyche ayura***

## Supplementary Material

XML Treatment for Amphoropsyche

XML Treatment for Amphoropsyche real
